# Comparison of traditional and upper thoracic epidural analgesia after off‐pump coronary artery bypass graft surgery: A Quasi‐experimental study

**DOI:** 10.1002/hsr2.774

**Published:** 2022-08-10

**Authors:** Sanjoy Kumar Saha, Redoy Ranjan, Asit Baran Adhikary

**Affiliations:** ^1^ Bangladesh University of Professionals Dhaka Bangladesh; ^2^ Bangabandhu Sheikh Mujib Medical University Dhaka Bangladesh; ^3^ Royal Holloway University of London Egham UK

**Keywords:** conventional analgesia, off‐pump coronary artery bypass graft, pain, thoracic epidural analgesia, VAS

## Abstract

**Background and Aims:**

Surgical trauma initiates changes in central and peripheral nervous systems that need to be treated therapeutically to facilitate postoperative pain. The quality of postoperative analgesia is expected to affect clinical outcomes positively. Albeit optimal pain relief following cardiac surgery is often complex, researchers have tried to explore several techniques other than conventional ones during the last decade to find a unique analgesic method for postcardiac surgical patients. This study aims to find a unique analgesic approach that maximizes patient satisfaction after off‐pump coronary artery bypass graft (OPCABG) surgery.

**Methods:**

The current study will compare the analgesic effect of upper thoracic epidural analgesia (TEA) with conventional analgesia after OPCAB graft surgery. For this, we will use a Quasi‐experimental study design. Patients admitted for coronary artery bypass graft (CABG) surgery will be assigned into two groups. The control group (conventional) will receive intravenous opioids and nonsteroidal anti‐inflammatory medications, and the study (case) group (TEA) will receive Inj. Bupivacaine 0.25% as an infusion through the epidural catheter. Physiologic parameters like hemodynamic and respiratory variables and pain scores will be recorded in predesigned format periodically.

**Results:**

We expect to analyze a total of 130 consecutive off‐pump CABG surgery patients in Group A (Case, 65 patients) and Group B (Control, 65 patients). Study variables will be the visual analog scale score, hemodynamic parameters (heart rate, mean arterial pressure, and respiratory parameters (respiratory rate, PaO_2_, PaCO_2_, PEFR, FEV_1_). After data collection, the result will be analyzed and published in the public domain and in journals.

**Conclusion:**

We expect thoracic epidural analgesia with local anesthetics will be a reliable postoperative analgesic option.

## 1 INTRODUCTION

Postoperative pain control improves clinical outcomes and patient satisfaction as well as lowers pain scores after cardiac surgery. The quality of postoperative analgesia positively affects morbidity in cardiac surgery.^1^ Thoracic epidural analgesia (TEA) is a valuable tool for acute pain relief for both anesthesiologists and surgeons. Hoar et al. first described the use of TEA for patients having heart surgery in the modern surgical era.^2^ Subsequently, thoracic epidural anesthesia and analgesia have been used to manage patients having cardiac, thoracic, and upper abdominal procedures during and after surgery.^3^, ^4^

Off‐pump coronary artery bypass graft (OPCABG) is a commonly practiced procedure for ischemic heart disease worldwide.^2^, ^3^, ^4^ For the first time in Bangladesh, Professor Asit Baran Adhikary and his team performed OPCAB surgery using epidural anesthesia on March 9, 2006 in a private hospital in Dhaka, which opened a new era on the horizon of cardiac surgery in Bangladesh which facilitated epidural analgesia in cardiac surgical patients.^5^

International Association for the Study of Pain (IASP) defined pain as “An unpleasant sensory and emotional experience associated with or resembling that associated with, actual, or potential tissue damage.”^6^ Postoperative pain stimulates the sympathetic nervous system and increases myocardial oxygen consumption. Oxygen supply‐demand mismatch can lead to myocardial ischemia, detrimental to patient outcomes.^7^, ^8^ Optimal postoperative pain management with a regular and routine assessment of pain will improve postoperative outcomes. The patient's assessment of pain using a visual analog scale (VAS) is the most reliable and should be used when possible.^9^

Traditionally opioids, nonsteroidal anti‐inflammatory medications (NSAIDs), or a combination of both, are being used to provide analgesia following cardiac surgery, which is linked to adverse side effects.^10^, ^11^ Longer‐acting opioids, such as morphine, delayed postoperative tracheal extubation due to drowsiness and respiratory depression. Concerns about adverse events NSAIDs include altered stomach mucosal barrier, renal tubular function, and platelet aggregation inhibition, limiting their usage in cardiac surgery.^12^

Despite many new methods, advanced pharmacological analgesic preparations, and proven pain services, postoperative pain control in coronary artery bypass graft (CABG) surgery has remained a challenge. As a general rule, single‐modality therapy is often recommended to treat immediate postoperative pain.^10^, ^11^, ^12^, ^13^ In the current era of early extubation (fast‐tracking), cardiac anesthesiologists are investigating novel options for adequate analgesia in patients undergoing cardiac surgery that are not traditional opioids and NSAIDs.^1^, ^14^

This current study will aim to evaluate an effective method of optimum postoperative analgesia using either TEA (Inj. Bupivacaine 0.25% at 3–5 ml/h continuous infusion) or conventional analgesia (intravenous Ketorolac 30 mg 08 hourly with or without narcotic analgesic) after OPCAB surgery. Furthermore, we also aim to evaluate postoperative cardiac and respiratory variables in relation to pain intensity after OPCAB surgery.

### 1.1 Research hypothesis

#### 1.1.1 Null hypothesis (H_0_)

There is no significant difference between conventional and upper TEA in pain control after OPCAB surgery.

#### 1.1.2 Alternative hypothesis (H_a_)

Upper TEA with Inj. Bupivacaine will provide better postoperative analgesia than conventional one after OPCAB surgery.

### 1.2 Research gap

Researchers explore significant pain control after cardiac surgery using different methods (e.g., local infiltration, intercostal and paravertebral blocks, spinal and epidurals, patient‐controlled analgesia) of pain services compared to conventional ones. Furthermore, an effective single modality of analgesia has yet not been established.

There is seldom a study performed in Bangladesh to explore the role of epidural analgesia after off‐pump CABG. So, the research gap is to find an effective analgesia method after OPCAB surgery using TEA. We characterize the research gap with the PICO framework as follows:

*Population* (*P*): Patients admitted for scheduled OPCAB surgery.

*Intervention* (*I*): TEA using Inj. 0.25% Bupivacaine as a continuous infusion pump.

*Comparison* (*C*): Traditionally used opioids and NSAIDs (Inj. Ketorolac 30 mg iv) analgesics.

*Outcomes* (*O*): Improved postoperative analgesia and reduced postoperative morbidity.

### 1.3 Conceptual framework

**Diagram 1:**
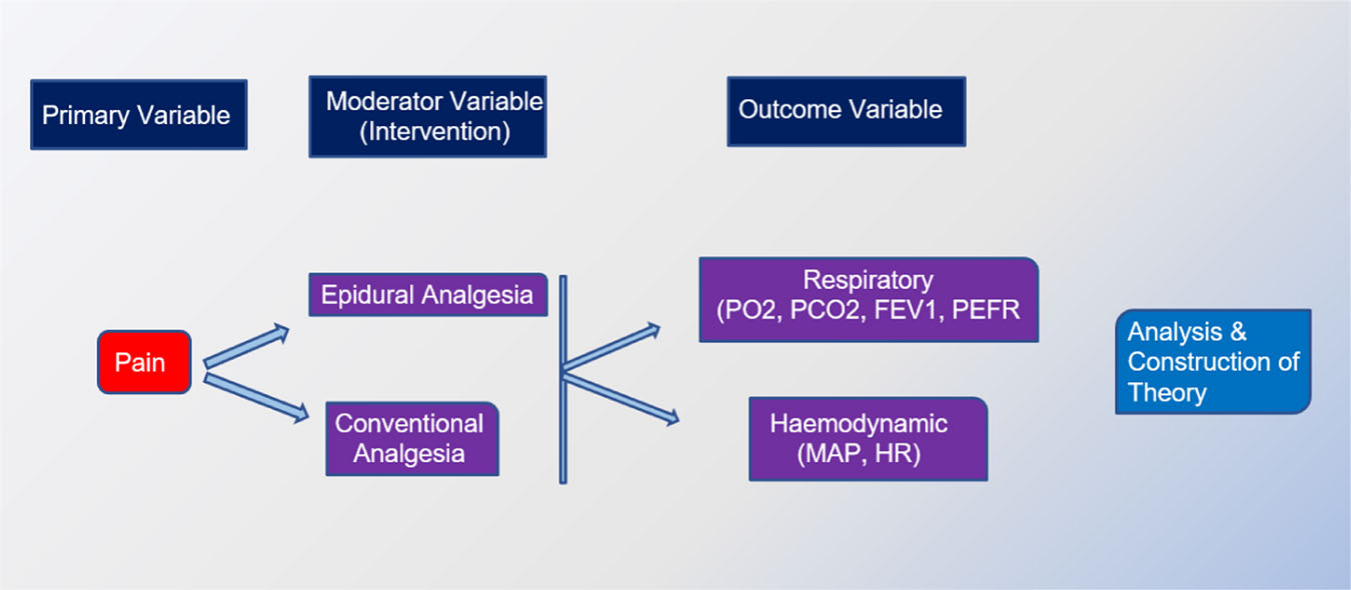
Research paradigm illustrating the steps of the current study protocol from population recruitment to final analysis and construction of hypothesis.

Early extubation and hemodynamic stability are the primary concerns of cardiac anesthesiologists in the modern era of fast‐tracking. The analytical framework (Diagram 1) for this study has been created based on the study's objective. The primary aim of this study is to find an efficient and reliable analgesic approach following OPCAB surgery.

## 2 PATIENTS AND METHODOLOGY

This Quasi‐experimental Study design will be conducted from February 2021 to July 2022 at the Department of Cardiac Surgery, Bangabandhu Sheikh Mujib Medical University, and Al‐Helal Specialized Heart Hospital, Bangladesh. Patients who will undergo elective off‐pump CABG surgery will be included in the study. The study exclusion criteria are patient refusal or uncooperative patient, abnormal coagulation profile, previous cardiothoracic, cervical, or upper thoracic operation, known case of chronic obstructive pulmonary disease, chronic kidney disease, chronic liver disease malignancy, systemic or local infection, and any spinal abnormality. Also, the patient who converted from OPCABG to On‐pump during the procedure will be excluded from the study.

A total of 130 patients will be evaluated in two groups; Group A (Case 65 patients): off‐pump CABG surgery under general endotracheal anesthesia and will receive postoperative TEA infusion (Inj. Bupivacaine 0.25%); and Group B (Control 65 patients): off‐pump CABG surgery under general endotracheal anesthesia and will receive postoperative conventional analgesia (intravenous Ketorolac 30 mg with or without narcotic analgesics). Study variables will be VAS score, hemodynamic parameters like heart rate, mean arterial pressure, and respiratory parameters (respiratory rate, partial pressure of oxygen_2_, partial pressure of carbon dioxide, peak expiratory flow rate, & forced expiratory volume in the 1st second.

In our study, a continuous response variable, pain score by VAS among two groups, TEA and conventional analgesia among OPCAB patients. In a previous study, within each subject group, responses were generally distributed with a 2.3 standard deviation.^14^ If the actual difference in the two group means of VAS score is 1.0; in that case, we will need to study 65 for TEA and 65 for conventional analgesia in OPCAB patients to reject the null hypothesis with a probability (power) of 0.8 that the population means of the two groups are equal. The probability of Type I error is 0.05 and *Z_α_* = 1.645 & *Z_β_* = 0.8416, (as H_a_: µ_a_ > µ_0_) for this null hypothesis test. Therefore, we will need 65 subjects in each group, and the total sample size will be at least 130.

$$n=\frac{2\sigma^{2}}{\Delta^{2}}{\left(Z_{\alpha }+Z_{\beta }\right)}^{2}$$

The above formula used to calculate the sample size is required to compare two groups when the endpoint is quantitative data.^15^, ^16^

### 2.1 Ethical consideration

Before starting data collection, informed written consent would be taken from all the respondents. The confidentiality of subjects will be protected by offering assurance to respondents that the information they provide will not be revealed and will only be used for study purposes. Patients will have the right to agree, disagree, or leave the study.

### 2.2 Statistical analysis

Statistical analysis will be conducted using Statistical Package for Social Sciences (SPSS) version 25.0 for windows software. The student's *t*‐test will evaluate the continuous data, and categorical data will be evaluated using the *χ* ^2^ test. A *p* ≤ 0.05 will be considered statistical significance.

## 3 OPERATIVE DEFINITIONS

### 3.1 VAS

**Figure 1:**
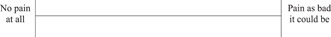
A 10 cm visual analog scale (VAS scale) marked as “no pain at all” and “pain as bad it could be,” and patients will be asked to point out the intensity of pain on the VAS scale.

A VAS is a tool for measuring the pain intensity, which is drawn as 10 cm of a straight horizontal line on a paper, labeled as “no pain at all” and “pain as bad it could be” at the other end^17^ (Figure 1). Subjects are asked to point out their pain intensity on the VAS scale. Pain is evaluated, and measurement is carried out as the distance between “no pain” to the point where the patient marks on the scale.

### 3.2 Off‐pump CABG

**Diagram 2:**
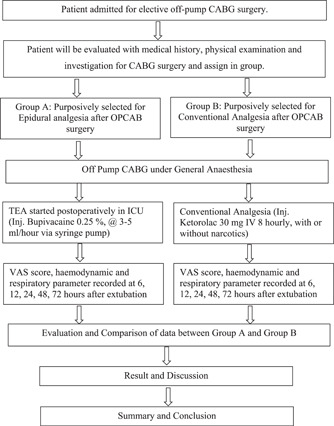
Illustrates flow chart of study procedure. CABG, Coronary artery bypass graft surgery; ICU, intensive care unit; OPCAB, off‐pump coronary artery bypass; TEA, thoracic epidural analgesia; VAS, visual analog scale.

The first left internal mammary artery anastomosis to obtuse marginal (OM) was conducted in St Petersburg in 1964 by Kolessov and colleagues without using the heart–lung machine, hence called OPCAB.^18^ Instead of stopping the heart, technical advancements and new types of surgical equipment (intracoronary shunt, epicardial stabilizer) allow the surgeon to hold and stabilize the heart throughout the operation (Diagram 2).

## 4 LITERATURE REVIEW

Surgical stress, pain, and breathing difficulties contribute to the morbidity and mortality associated with heart surgery. Following cardiac surgery, pain disrupts the humoral and neuroendocrine systems' equilibrium, increasing stress hormone levels and myocardial oxygen demand.^5^, ^6^, ^7^, ^8^, ^9^, ^10^, ^11^, ^12^ As a result, arrhythmias and myocardial ischemia are increased. Pain also impairs mechanical ventilation and mobility restriction.^2^, ^10^, ^11^, ^12^, ^13^, ^14^, ^15^ Postoperative pain management in cardiac surgery has traditionally relied on pharmacologic therapy, either parenteral opioids or NSAIDs.^16^, ^17^, ^18^, ^19^, ^20^ However, they rarely provide analgesia comparable to a regional anesthetic.^21^ CABG surgery can be performed using a variety of regional anesthetic approaches. Thoracic epidural anesthesia should be preferred for thoracic surgeries due to its longer duration and advantage of prolonged postoperative pain management.^22^ However, the use of TEA decreased the incidence of myocardial infarction (MI), and it enhanced left ventricle (LV) function in patients undergoing aortic valve replacement alone or in combination with CABG, even when the left ventricular mass was increased.^23^

Cardiac surgery requires anticoagulants, and it is not widely accepted due to concerns about neuraxial hematoma following anticoagulants.^1^, ^14^, ^21^, ^22^, ^23^, ^24^, ^25^, ^26^ A few trials have demonstrated the safety of regional anesthetic and analgesic techniques in the context of possible anticoagulation during cardiac surgery.^1^, ^19^ Regional blocks have enormous potential to be integrated into hospitals' acute pain management paradigms following cardiac surgery, given their initial success in decreasing pain scores and reducing opiate usage.^26^

Spinal and epidural instrumentation entails risk, with epidural hematoma formation being the most feared complication.^25^, ^26^, ^27^, ^28^ Patients who did not receive thromboprophylaxis have a hematoma formation rate of one in every 220,000 spinal anesthesia cases and one in every 150,000 epidural anesthesia cases but in cardiac surgery, it is approximately 1:3552 observed by Landoni and colleagues.^27^, ^28^, ^29^ The VAS is frequently used to explore various subjective experiences, including pain. Subjects are asked to demonstrate severity by marking a 10 cm long horizontal line with the words “no pain” at one end and “worst pain conceivable” at the other end with a VAS. The essential advantage of VAS is that the ratings have the characteristics of ratio data and may be analyzed objectively as such, providing that the data are generally distributed. The VAS is applicable in a wide variety of clinical and testing situations.^30^, ^31^

Prospective, randomized research on postoperative pain following coronary artery bypass was conducted in Istanbul to determine the effects of thoracic epidural as an adjunct to general anesthesia. In the early postoperative phase after the CABG, TEA substantially decreased the severity of postoperative pain and the use of pain analgesics.^30^, ^31^, ^32^ Cardiac sympathectomy induced by TEA at spinal levels T1–T5 increases coronary flow and the balance of myocardial oxygen delivery and consumption and flow through the internal thoracic artery, the most commonly used conduit for coronary revascularization.^33^ Providing adequate perioperative analgesia has been shown to improve hemodynamics and immunologic responses, which may lead to improved outcomes after heart surgery.^26^

Thoracic epidural anesthesia combined with general anesthesia during OPCAB decreases the need for mechanical ventilation following surgery, decreases the length of stay in the intensive care unit (ICU), and provides better pain control than general anesthesia alone.^30^, ^31^, ^32^, ^33^ Furthermore, in a recent study, Mehta et al.^34^ observed combined efficacy of general anesthesia with TEA and observed better postoperative analgesia and clinical outcomes, especially cardiorespiratory and renal functions, and allowed first tract extubation following high‐risk cardiac surgery, similar to another European study findings conducted by Stenger et al.^35^ in Denmark. In this study, the authors aim to justify that the use of TEA shortens mechanical ventilation time and return of normal respiratory function, ICU stays, and provides stable hemodynamics compared to general anesthesia alone.^30^, ^31^, ^32^, ^33^, ^34^, ^35^

## 5 SIGNIFICANCE OF THE STUDY

Pain after cardiac surgery stimulates a stress response, which has the potential to cause pathophysiological changes in all of the body's major organ systems. Satisfactory postoperative analgesia is associated with a lowered stress response and thus reduces patient discomfort and may reduce morbidity who have undergone OPCAB surgery.

In general, single‐modality therapy is the most effective treatment for acute postoperative pain, and it is expected that the TEA (Group A) will be beneficial with significant postoperative pain relief and will show better hemodynamic and respiratory parameters.

## 6 CONCLUDING REMARKS

Patients' satisfaction with the quality of postoperative analgesia is closely linked to the comparison between predicted and perceived pain. The adequacy of postoperative pain relief remains a concern for patients undergoing OPCAB surgery. As postoperative pain relief is crucial after off‐pump CABG, considering all other modalities, upper TEA may be the choice of postoperative analgesia.

## AUTHOR CONTRIBUTIONS

**Sanjoy Kumar Saha**: Conceptualization; data curation; methodology; resources; validation; writing–original draft; writing–review & editing. **Redoy Ranjan**: Conceptualization; data curation; formal analysis; methodology; resources; writing–original draft; writing–review & editing. **Asit Baran Adhikary**: Conceptualization; methodology; supervision; validation; writing–review & editing. All authors have read and approved the final version of the manuscript.

## CONFLICT OF INTEREST

The authors declare no conflict of interest.

## TRANSPARENCY STATEMENT

Professor Dr. Asit Baran Adhikary affirms that this manuscript is an honest, accurate, and transparent account of the study being reported, that no important aspects of the study have been omitted, and that any discrepancies from the study as planned (and, if relevant, registered) have been explained.

## Data Availability

This is an ongoing project and the data that support the findings of this study are available on request from the corresponding author. The data are not publicly available due to privacy or ethical restrictions. Professor Dr. Asit Baran Adhikary had full access to all of the data in this study and took complete responsibility for the integrity of the data and the accuracy of the data analysis.
